# New Electromagnetic Interference Shielding Materials: Biochars, Scaffolds, Rare Earth, and Ferrite-Based Materials

**DOI:** 10.3390/nano15070541

**Published:** 2025-04-02

**Authors:** Dragana Marinković, Slađana Dorontić, Dejan Kepić, Kamel Haddadi, Muhammad Yasir, Blaž Nardin, Svetlana Jovanović

**Affiliations:** 1 Vinča Institute of Nuclear Sciences, National Institute of the Republic of Serbia, University of Belgrade, P.O. Box 522, 11001 Belgrade, Serbia; sladjana.dorontic@vin.bg.ac.rs (S.D.); d.kepic@vin.bg.ac.rs (D.K.); 2 University of Lille, CNRS, Centrale Lille, University Polytechnique Hauts-de-France, UMR 8520-IEMN-Institut d’Electronique de Microélectronique et de Nanotechnologie–Lille, 59650 Villeneuve-d’Ascq, France; kamel.haddadi@univ-lille.fr; 3 Department of Computer Science, Division of Microrobotics and Control Engineering, University of Oldenburg, Ammerländer Heerstraße 114-118, 26129 Oldenburg, Germany; muhammad.yasir@uni-oldenburg.de; 4 Faculty of Polymer Technology, Ozare 19, 2380 Slovenj Gradec, Slovenia; blaz.nardin@ftpo.eu

**Keywords:** EMI shielding materials, mechanisms of EMI shielding, biochars, scaffolds, rare earth, ferrite-based materials

## Abstract

In this review, a comprehensive systematic study of the research background, developments, classification, trends, and advances over the past few years in research on new electromagnetic interference (EMI) shielding materials will be described. The following groups of new materials for EMI shielding will be discussed: biochars, scaffolds, rare earth, and ferrite-based materials. We selected two novel, organic, lightweight materials (biochars and scaffolds) and compared their shielding effectiveness to inorganic materials (ferrite and rare earth materials). This article will broadly discuss the EMI shielding performance, the basic principles of EMI shielding, the preparation methods of selected materials, and their application prospects. Biochars are promising, eco-friendly, sustainable, and renewable materials that can be potentially used as a filter in polymer composites for EMI shielding, along with scaffolds. Scaffolds are new-generation, easy-to-manufacture materials with excellent EMI shielding performance. Rare earth (RE) plays an important role in developing high-performance electromagnetic wave absorption materials due to the unique electronic shell configurations and higher ionic radii of RE elements. Ferrite-based materials are often combined with other components to achieve enhanced EMI shielding, mechanical strength, and electrical and thermal conductivity. Finally, the current challenges and future outlook of new EMI shielding materials will be highlighted in the hope of obtaining guidelines for their future development and application.

## 1. Introduction

The human population in urban and rural regions is exposed to non-ionizing electromagnetic fields (EMFs) due to the rapid development of telecommunication technology and digital systems for information transfer [1,2]. The association of the fifth generation (5G) and upcoming sixth generation (6G) will lead to the emergence of innovative applications (Internet of Things and self-driving vehicles) [3,4]. Wearable and portable electronics will soon become more commonly used [4]. The COVID-19 pandemic triggered the implementation of technology trends such as digital payments, telemedicine, and robotics, which use radiofrequency (RF) electromagnetic waves (EMWs) in the 100 kHz–300 GHz frequency range [5]. These technological advances caused an increase in artificial sources of EMF and resulted in the chronic exposure of people and the environment, creating electromagnetic pollution. Electromagnetic pollution is the continuous and uncontrolled exposure to electromagnetic fields from any emitting source of the EMF spectrum [5]. From 1950 to 2010, the levels of exposure to RF-EMF have increased from extremely low natural levels by about 1018 times [6]. Many studies have shown the harmful effects of EMF on human health [7,8,9,10,11] and the environment [12,13,14,15]. In addition to the potential health risks, the interference of EMW worsens heat accumulation, significantly shortening equipment lifespan [16]. Thus, new electromagnetic interference (EMI) shielding materials are urgently needed.

The conventional EMI shielding materials are metals, carbons, ceramics, cement, conductive polymers, and associated composites. Metals and carbons are the most functional materials due to their high conductivity, while ceramics, cement, and (non-conductive) polymers are less efficient unless combined with functional components/materials [17,18]. Shielding barriers should meet the required dimensions and shapes at the lowest possible cost for various applications.

Among the various new materials, organic materials such as graphene, carbon black, and carbon nanotubes are highly conducting, lightweight, flexible, and highly efficient EMI shielding materials. Still, their high price and the complexity of production and purification lead to high cost and limit their use. Inorganic materials, such as metal powders and metal oxides, are highly efficient EMI shielding materials due to their high electrical conductivity accompanied by magnetic permeability. Challenges in their application are associated with their high prices, chemical instability, corrosion, and heavy weight. Thus, in this review paper, we are focusing on new, suitable, low-cost organic materials—biochars and scaffolds. These organic EMI shielding materials are compared to novel inorganic shielding materials—rare earth and ferrite-based composites. By reviewing their production methods, yields, physico-chemical properties, and EMI shielding effectiveness, the future direction in the development of EMI shielding materials is estimated.

According to the reviewed literature, biochars are promising, eco-friendly, sustainable, and renewable materials that can potentially be used as filters in polymer composites for EMI shielding applications in electronic devices and construction materials. Previous research showed that scaffolds are new-generation, easy-to-manufacture materials with excellent EMI shielding performance.

Recently, great attention has been paid to traditional magnetic materials/alloys with excellent absorption of EMWs, but they are often limited by thickness and range of use. However, according to Snoek’s limit, where the direct current (DC) susceptibility and the cut-off frequency are constant, the permeability value decreases in the high-frequency range, which leads to an apparent decrease in magnetic loss capability [19,20,21]. Improving the permeability and magnetic loss of magnetic materials/alloys at high frequencies is crucial for obtaining good electromagnetic/microwave absorption properties [22]. It was shown that the electronic structure and well-chosen methods for designing magnetic materials/alloys can improve magnetism at high frequencies [23].

Therefore, magnetic metal oxide composites or perovskite oxides doped with rare earth element ions have been in development for the last few years due to their specific structures, interfacial polarization, multiple reflections, and excellent conducting and EMI shielding properties [24].

Rare earth materials are often characterized by various names, such as rare earth elements (REEs), rare earth metals (REMs), rare earth oxides, or yttrium-based rare earth material. This group represents a set of 17 chemical elements, as listed in the periodic table, from lanthanum (La) to lutetium (Lu), including 15 lanthanides and scandium (Sc) and yttrium (Y) with a tendency to occur in the same core deposits as lanthanides and exhibiting identical chemical properties [25]. In the crystal structure, REEs are most often found in the trivalent state (RE^3+^), although the divalent state (RE^2+^) is also possible. REEs with strong inter-electron interactions with their localized 3d and 4f electrons as well as strong coupling with magnetic materials have emerged as outstanding material dopants [26]. REEs based on neodymium (Nd), dysprosium (Dy), and samarium (Sm), due to their relatively large number of unpaired electrons in the atomic structure, possess a high remanent magnetization and coercivity value determining the stability of the remanent state. The orbital electron structure of these elements contains many unpaired electrons, which allows them to store large amounts of magnetic energy. The total magnetic moment originates from these unpaired electrons [27,28]. The reviewed literature revealed the growing interest in rare-earth-based materials as materials for EMI shielding applications [29,30]. These materials can be classified into several groups: RE-doped ferrites (RE-Fs), F_2_O_3_/Fe_3_O_4_, RE–transition metal intermetallics (RETMIs), RE oxides (REOs), RE-based alloys, RE spinels, RE-doped MoS_2_, and RE polymers. The above-mentioned groups of rare-earth-based materials will be discussed in detail throughout this manuscript. The main advantages of rare-earth-based materials over traditional EMI shielding materials are the magneto-dielectric effects and tunable dielectric properties in an electric or magnetic field. These properties make them attractive for exploring their EMI shielding applications.

New nanocomposite-based foams and heterogeneous layered structures have shown outstanding EMI shielding properties. Namely, thermoplastic polyether-block-amide elastomer beads coated with Ti_3_C_2_T_x_ showed an EMI shielding efficiency of 44 dB at 8.5–12.5 GHz [29], microcellular aramid nanofiber/Ti_3_C_2_T_x_ MXene foams reached a value of 64.9 dB [30], and foams based on the conductive polymer polypyrrole with Fe_3_O_4_ achieved a value of 41.1 dB [31]. Combining MXenes with carbon nanotubes and silver nanowires in a layer-by-layer architecture, Zhang et al. achieved an EMI shielding efficiency of 53.1 dB in the X-band [32].

**Figure 1:**
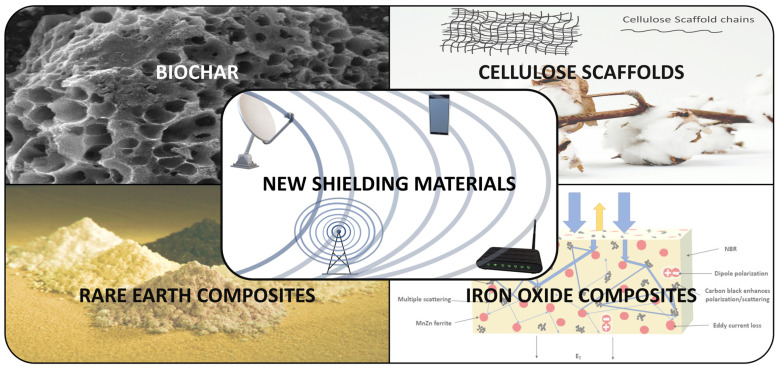
Schematic representation of new shielding materials based on biochars, scaffolds, rare earth, and ferrite materials discussed in this review. Part of Figure 1 (lower, left part) is adapted from reference [33].

The development, classification, trends, and advancements of research on novel shielding materials in electromagnetic irradiation during the past few years will be thoroughly and methodically reviewed herein. The recent progress in the development of new-generation shielding materials based on biochars, 3D network structures (referred to as scaffolds in the following), materials with rare earth (referred to as rare-earth-based materials in the following), and ferrite materials will be described (Figure 1). This paper will comprehensively discuss the EMI shielding performance, basic principles and mechanisms, preparation methods, structure, morphology, and application prospects of these materials.

## 2. New EMI Shielding Materials: Biochars, Scaffolds, Rare Earth, and Ferrite-Based Materials

### 2.1. Biochar as EMI Shielding Material

Among conventional EMI shielding materials, metals such as Cu, Ag, Al, etc., are highly efficient EMI shielding materials due to their electrical conductivity and ability to reflect incident EMWs [16]. Regardless, the use of these metals is limited owing to their high density, inconvenient processing, and poor resistance to corrosion [16]. Recently, shielding materials have been investigated in the form of composites with plastic or metals as substrates [34]. As alternative solutions, conductive polymer-based composites, porous conductive foams, and polymers with micro- and nanoscale filters such as carbon material (graphite, graphene, reduced graphene oxide, carbon nanotubes), MXenes, metal nanoparticles, and nanowires have been explored due to their low mass densities, good flexibility, and stretchability [4,35].

**Figure 2:**
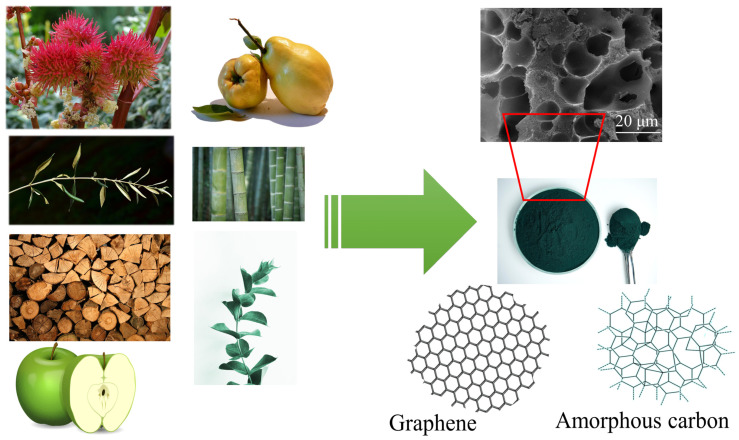
Different starting materials (*Ricinus communis* outer shell, bamboo, wood, apple, and quince stillage, olive tree pruning, eucalyptus) in biochar production, at various temperatures, usually in an oxygen-controlled atmosphere, lead to the production of porous materials with domains with graphene and amorphous carbon.

One of the latest approaches in developing new, sustainable, and eco-friendly solutions for EMI shielding is the integration of natural, renewable carbon materials—biochars—into polymers [4]. Biochars originate from various biomass sources, such as bamboo, sugarcane, and cork. To create biochars from biomass, biomass resources are converted to carbon materials by pyrolytic processes in an oxygen-limited environment. A schematic presentation of biochar starting materials and production steps is displayed in Figure 2. After high-temperature carbonization, biochars show extraordinary hardness, excellent thermal stability, and high electrical conductivity, making them suitable fillers for polymers [36]. By combining biochars and polymers, the mechanical, physical, and electrical properties of both materials can be upgraded successfully.

The electrical conductivity of biochar is closely related to the source properties and carbonization conditions [37]. Nevertheless, some researchers have disregarded the electrical properties of biochars, so their potential application in EMI shielding has rarely been examined [38,39,40].

In 2018, Li et al. conducted an initial research study [39]. Namely, they used commercially available bamboo charcoal as the biochar filler and/or an ultra-high-molecular-weight polyethylene (UHMWPE)/linear low-density polyethylene (LLDPE) blend as a potential EMI shielding composite. The composite with 80 wt.% of biochar was fabricated using the mass-producing extrusion and hot compression methods. Through the carbonization of bamboo charcoal at 1100 °C, a graphitic-like structure with good electrical conductivity and a high specific surface area was obtained. Namely, an electrical conductivity of 107.6 S/m and EMI shielding effectivity (SE) of 48.7 dB were measured at 1.5 GHz. The specific EMI SE of the composite was 39.0 dB cm^3^/g. This value is four times higher than the specific EMI SE of copper (10.0 dB cm^3^/g) [41].

Tolvanen et al. applied the same approach to prepare a biodegradable two- and three-phase composite of graphite, biochar derived from pine chips, and polylactic acid (PLA) [4]. The EMI shielding performances were tested in the K-band (18–26.5 GHz). The composite showed an outstanding EMI SE of >32 dB when the materials were prepared in the form of a film with a thickness of 25 μm and a high specific shielding effectiveness (SSE/t) of >890 dB cm^2^/g. It can be seen that these composites are convenient for the application of wearable/portable and stationary devices.

Shortly after, Akgül et al. presented a biochar–iron (BC-Fe) material obtained by the pyrolysis of industrial tea waste biomass and the encapsulation of BC-Fe into polymethyl methacrylate (PMMA) polymer [42]. During synthesis, Fe(III) ions promoted the graphitization of the amorphous carbon in biomass and contributed to biochar surface stabilization. The results of EMI SE measurements showed that the reflection component of the EMI SE of pure PMMA was near −10 dB at frequencies of 7.8 and 10.1 GHz, while it was reduced by ~60% when the content of BC-Fe in the polymer was 40 wt.% in the frequency range of 8.5–12.9 GHz.

Liang et al. successfully developed a mortar/brick structure using wood-derived porous carbon (WPC) as a skeleton and conductive 3D MXene aerogel [1]. The MXene aerogel/WPC composite was composed of highly ordered honeycomb cells inside WPC as a microreactor. Herein, a high graphitization level of natural wood was achieved by applying a high carbonization temperature of 1500 °C. The MXene aerogel/WPC composite showed an excellent EMI SE value of 71.3 dB in a frequency range of between 8.5 GHz and 12.5 GHz, while the sample density was only 0.197 g/cm^3^. In the study, a wall-like mortar brick structure (WPC as mortar and MXene aerogel as brick) solved the instability of the MXene aerogel network as well as prolonged the transmission path of EM waves, dissipating the incident EM waves in the form of heat and electrical energy. The material’s superior EMI shielding performance was achieved thanks to a specific design.

The shielding efficiency of a gypsum–biochar drywall-like composite was investigated by Natalio et al. [43]. They used wooden chips and eucalyptus biochar and combined them with gypsum. An enhancement in the shielding efficiency was recorded with an increased biochar content in the composites. Namely, the EMI SE values of the drywall-like plates with 10%, 20%, and 40% *w*/*w* biochar contents were 11.65 ± 1.6, 19.2 ± 5.7, and 19.25 ± 1.8 dB at a frequency of 6 GHz. This investigation contributes to expanding innovative bio-based sustainable materials with EM shielding properties in the microwave region.

Savi et al. used sewage sludge to obtain biochar and investigated its EMI-shielding features [44,45]. The biochar was mixed with epoxy polymer (20 wt%) and cast into a film 4 mm thick. The composite showed a promising EMI SE of −10 dB [44]. Savi et al. reported an outstanding electrical conductivity of 300 S/m [44]. The same group compared the EMI SE properties of biochar produced from carbonized sludge with graphene nanoplatelets [46]. Herein, the authors produced polymer composites with polyvinylidene fluoride (PVDF), while fillers (biochar and graphene) were added at 90 wt%. The biochar–PVDF composite showed transmission scattering of less than −30 dB.

Savi et al. also studied commercial biochar produced from wood biomass to coat several layers of common building components, such as drywall panels [47]. They recorded EMI SE values of 17 dB at 1 GHz and 25 dB at 18 GHz. Drywall panels coated with several layers of biochar are easy to fabricate and a low-cost solution to realize a protected surrounding for healthcare applications (chemotherapy and tomography) to minimize the intensity of the EM field close to electronic equipment. The same research group produced a cement-based composite with commercial lignin-based biochar and polyvinyl chloride (PVC) [48]. They introduced biochar powder into cement paste to improve its shielding properties. PVC, which was used as a filler, was obtained by decommissioning old electrical cables that would have ended up in a landfill. A combination of 10 wt.% biochar and 6 wt.% PVC revealed the best shielding capacity, around 16 dB in the 5.4–8 GHz frequency range. This investigation shows great importance in the EMI shielding application and from the aspect of the circular economy.

Miao et al. fabricated a conductive EBC@CNF@MWCNT composite aerogel by the freeze-drying process, mixing ferrite chloride with electrically conductive bamboo charcoal (EBC), with cellulose nanofibrils (CNFs) as a skeleton and multi-walled carbon nanotubes (MWCNTs) as a conductive enhancer in the freeze-drying process [36]. Afterward, the aerogel was soaked with polydimethylsiloxane and hot-pressed into the membrane. In the composite, EBC and MWCNTs were arranged uniformly in the CNF skeleton to form a 3D conducting network. The composite displayed exceptional electrical conductivity (47 S/m) and a high EMI shielding effectiveness of 39.5 dB, with an adsorption loss of ~75%.

Recently, Nikolopoulus et al. used biochar prepared from olive tree pruning to fabricate composite samples with carbon black and polytetrafluoroethylene as binders [49]. The EMI shielding capacity was measured in the 1–3 GHz frequency range. The results demonstrated that the raw pure biochar had a low EMI SE, between 1.5 and 4 dB, which was enhanced with an increase in the thickness from 0.1 to 0.5 mm. On the contrary, the composite significantly improved the EMI SE, reaching a value of 39 dB. This work indicates that biochar could be used as a basis for developing composites with high EMI SE values.

Milenkovic et al. recently reported that agricultural biowaste collected after apple and quince processing could be converted into EMI shielding material [50]. The starting materials were chopped, homogenized, dried, and mixed with an equal mass of KOH. The mixtures were carbonized at 850 °C, under nitrogen flow. The resulting biochars were mixed with sodium silicate at 40 wt.%. The film of only 0.2 mm thickness showed an EMI SE of 15.5 dB in the 8–12 GHz frequency range. Although moderate EMI SE values were found, the study demonstrates the outstanding potential of selected biowaste in the production of sustainable EMI shielding materials.

Another study by Perumal et al. proposed a new organic waste for biochar production [37]. Within the study, the composite built from *Ricinus communis* outer shell-based biochar and epoxy was fabricated by slow pyrolysis at 400–700 °C. The results showed that the biochar pyrolysis at 700 °C led to a maximal electrical conductivity of 95 S/m due to the presence of graphitic carbon. A maximum EMI SE of 26.5 dB was found in the X-band frequency range, at 40 wt.% biochar to epoxy matrix.

According to the reviewed literature, biochar is a promising, eco-friendly, sustainable, and renewable material that could be used as a filter for EMI shielding applications in electronic devices and construction materials. The biochar shielding effectiveness is associated with the electrically conductive graphene region in biochars and porous structure [50]. Namely, EMW attenuation is the result of wave reflection from the graphene region and multiple reflections inside the pores. Biochar features rely on two main factors:

(1) The experimental conditions (temperature, duration of heating, addition of chemical agent for pore formation or activitvation of graphitization, selected gas);
(2) The starting material’s chemical composition [39].

With an increased temperature, the porosity, specific free space, and carbon content increase too, while the selection of source materials affects both the properties and yields of the resulting biochar [39].

### 2.2. Scaffolds as EMI Shielding Material

Many structural patterns, including highly porous materials and conductive structures such as multilayer composites obtained by stacking cellulose nanofibers/reduced graphene oxide (rGO), rGO films, and multistage composite foams, are recognized as excellent EMI shielding materials. These structures are obtained using vacuum-assisted filtration, freeze-drying techniques, or electroless deposition [51]. The mentioned procedures allow for a reduction in the reflection coefficient, but they are complex and often not reliable. The innovative manufacturing technologies imply 3D network structures (scaffolds) that can be decorated with metal nanoparticles [52], coated by polymers, encapsulated with paraffin [53], etc. These approaches enable design freedom, flexibility, precise control over the shape and size, and the connectivity of porous structures.

Cellulose scaffolds (CSs) are recognized as a promising material for EMI shielding applications due to the ability of cellulose nanofibers to improve the thermal and mechanical features of nanocomposites. Tran et al. represented a 3D network structure of cellulose scaffolds decorated with silver nanoparticles (AgNPs) [52]. The scaffolds were filtered at the epoxy matrix and cured at 40 °C to fix the nanoparticles. The obtained composite possesses a thermal conductivity of 2.52 W/m/K, which is over 11-fold the thermal conductivity of pure epoxy. The extremely high electrical conductivity of 53.691 S/m caused a remarkable SE value of 69.1 dB. He et al. designed a scaffold connected with metal nanoparticles [53]. In this work, the shape-stable composite was composed of a paraffin impregnated in a biological porous carbon scaffold, followed by a coating with polyurethane and Fe_3_O_4_ nanoparticles. The biological porous carbon was obtained from a loofah sponge by immersion in a phenolic resin solution followed by carbonization. Scanning electron microscopy (SEM) results revealed that the system kept its original shape with a 3D and honeycomb-like porous structure of single fibers after carbonization. The polyurethane coating provided adequate mechanical support and effective leak-proof performance. The produced framework shows strong EMI shielding performance (up to 32 dB). Another study based on the encapsulation of Fe_3_O_4_ nanoparticles in carbon scaffolds was conducted by Wei et al. [54]. Herein, laminar cellulose-paper-based scaffolds with bidirectional gradient distributed Fe_3_O_4_ nanoparticles were constructed via immersion, drying, and carbonization processes. The resulting carbon scaffolds exhibited high in-plane electrical conductivity (96.3 S/m) and high shielding efficiency (1805.9 dB/cm^2^g).

A 3D cellulose scaffold was combined with a CNT/MXene composite [55]. Nanosheets of the composite were inserted into a cellulose scaffold by vacuum impregnation. Finally, a hydrophobic and multifunctional 3D system was produced by wrapping it with poly (dimethylsiloxane). The composite had a high compressive strength of 1.53 MPa, a maximum strain at fracture of 74.1%, and an outstanding SE (29.3 dB).

Hu et al. reported a dual-ice templating assembly strategy to prepare a dual-interpenetrated scaffold [56]. The scaffold was linked with a high-quality graphene array and porous MXene-Co aerogel. It showed an absorption-dominated EMI SE of 72.86 dB.

Three-dimensional-printing technology was applied for scaffold fabrication based on a triply periodic minimal surface and 70% porosity [42]. The monolithic layered dipping method was used to regulate the gradient distribution of carbon nanotubes on the 3D-printed scaffold surface and ensure integrity. This system blocked 99.9% of EM waves. The value of the SE in this case was 35.9 dB.

Previous research showed that scaffolds are new-generation, easy-to-manufacture materials with excellent EMI shielding performance. Due to their exceptional three-dimensional construction and inner network, they show anisotropic thermal conductivity and allow heat dissipation, making them efficient EMI shielding materials [52,56]. The results from the literature based on biochar and scaffolds in shielding applications are listed in Table 1.

### 2.3. Rare-Earth-Based Materials as EMI Shielding Material

Next-generation microwave-absorbing compounds and hybrid materials could potentially outperform traditional materials in terms of their ability to absorb microwaves, their persistence, their stability at high temperatures, weight reduction, their corrosion resistance, and the need to expand the frequency spectrum over which materials can efficiently absorb microwaves. Research and development in the last few years have promoted magnetic metal oxide composites or perovskite oxides doped with REEs as innovative materials for good electromagnetic wave absorbance due to their specific structures, interfacial polarization, and multiple reflections, showing excellent conductive properties and magnetic losses [60]. By doping rare earth elements, the dielectric loss and dipole polarization can be increased, and the wave absorption performance of ferrite can be further improved. Nikzad et al. [61] prepared a Nd^3+^-substituted Gd–Co ferrite composite and found this to be a promising EW absorption material with a wide effective absorption bandwidth in the X-band. On the other hand, it was observed that appropriate La doping enhances the dielectric and conductive mismatch levels of the material. This enhancement leads to a stronger interface polarization capability [62].

Different microstructures and morphologies manifest different EMI shielding properties. Addressing the challenge of creating absorption-dominant EMI shielding requires significant attention to detailed structural design [63]. This strategy aims to produce structures that minimize the reflection of undesirable electromagnetic noise, capturing electromagnetic waves (EMWs) through various attenuation mechanisms.

The rare earths (REs) for electromagnetic wave absorption applications can be classified into several groups: RE-doped ferrites (RE-Fs), RE–transition metal intermetallics (RETMIs), RE oxides (REOs), and other categories [64]. Graphene-based materials, metal–organic frameworks (MOFs), ferrites, molybdenum disulfide, MoS_2_, and other innovative materials can significantly enhance EMW absorption properties by doping or substitution with REs due to the magnetic moment originating from their unpaired electrons [65]. The high-frequency permittivity of rare-earth Er-doped MoS_2_ films was studied [66]. The size of nanocrystals depends on the reaction time; the reactant concentration and composition control the morphology [67]. With a lowered concentration, the resulting nanoparticles exhibited a spherical morphology but gradually changed to flower-shaped with increasing concentration.

Pure MoS_2_ has a relatively homogeneous loss mechanism, limited impedance matching, and a limited ability to absorb EW. Doping is an effective method to improve the electromagnetic properties of materials [68]. For instance, palladium-doped few-layer MoS_2_ nanosheets grown on the surface of multi-walled CNTs and the 10% Pd nanohybrid possess a higher EMI shielding effectiveness (SE ∼26.50 dB) than that of SE ∼21.55 dB in the undoped MoS_2_/CNT at the same thickness of 1 mm [69].

Laser-cladded novel FeCo-based alloys with different RROs, such as La_2_O_3_, Y_2_O_3_, Nd_2_O_3_, and Gd, possess enhanced electromagnetic shielding performance with a shielding effectiveness (SE) of up to ~96 dB at 23.9 GHz and an ability to absorb 99.9999% of EMWs [70,71].

Li et al. [72] discovered that REE substitution in Fe_4_N could modulate magnetic moments and magnetic exchange coupling interactions and the change in spin polarizability. Zhang et al. [73] studied the microwave absorption properties of high-entropy hexaborides containing rare earth, including Ce, Y, Sm, Er, and Yb, prepared by a boron carbide reduction reaction, which showed good absorption properties with an effective bandwidth of 4.3 GHz, while Chen et al. [74] studied the absorption properties of high-entropy rare earth silicide carbides and high-entropy rare earth oxides based on different ratios of Tm, Y, Dy, Gd, Tb, Pr, and Tb ions, prepared by a solid-state reaction method, which showed good absorption properties with an effective bandwidth of 4.5 GHz. Absorbent, magnetic cores made of alloys based on Fe and Nb are suitable for EMI reduction signals with a 6–7 MHz frequency. However, Zamborszky et al. constructed a broadband coaxial probe head allowing the measurement of Fe-based nanocrystalline cores containing Nb up to 1 GHz using a reflectometry method with a vector network analyzer (VNA) [75].

The effects of the 4f-3d interaction on electronic locality, magnetism, and charge migration during hybridization between Er and Fe/Co were studied [22,76]. Due to enhanced EMI shielding properties, La, Ce, Pr, Gd texaphyrin, Tb, and Er were studied and selected as potential new shielding materials, as well as Co–Mg–La ferrite/graphene composites [77,78,79]. Gd^3+^-doped MnFe_2_O_4_ synthesized by the sol–gel method expressing very low dielectric loss, saturation, and minimum coercivity can be a potential material with applications in storage and microwave absorption devices [80]. Gd-doped Fe_3_O_4_ oxide (Gd_x_Fe_3−x_O_4_) samples with different percentages of Gd contents were prepared by the hydrothermal method [81].

The substitution of smaller Fe ions with RE ions leads to significant changes in physical properties such as the structural distortion of crystal units, the formation of a new phase, and changes in the morphological, optical, electrical, and dielectric properties due to the different ionic radii between ions.

The partial substitution of RE ions, such as with Sm, Ce, Er, Dy, La, and Nd for Fe^3+^, leads to a structural bend in the spinel structure which induces strain and considerably modifies the electrical and dielectric properties [82].

All ferrites exhibit a cubic spinel structure with the Fd-3m space group. The ionic radius of RE^3+^ ions in this environment (Gd^3+^ = 0.94 Å, Eu^3+^ = 0.95 Å, Sm^3+^ = 0.96 Å, Nd^3+^ = 0.96 Å, Pr^3+^ = 0.99 Å) is larger than that of Fe^2+^ ions (0.65 Å) [83]. The substitution of Fe^2+^ by RE^3+^ ions limits solubility in the lattice and inhibits grain growth. The shifting of the positions of diffraction peaks to smaller two-theta angles with an increase in the RE^3+^ concentration results from the increased lattice constant [84,85,86].

RE substitution has been reported to immensely affect cobalt ferrite’s structural, morphological, and physical properties [87,88]. It induces strain, causes the distortion of the crystal lattice, promotes the creation of vacancies, reduces crystallite sizes, increases grain–grain boundary interfaces, and promotes nanoscale magnetic effects such as spin canting and the core–shell effect. The insertion of RE in place of Fe promotes 3d–4f coupling, leading to variation in the magnetic, dielectric, and conduction mechanisms, and an enhancement in the SE can be obtained.

RE substitution can also decrease the grain size, which is an important factor in low-noise media. Cheng et al. obtained an increase in the polar Kerr rotation for Er- and Tm-doped cobalt ferrite films, while no significant changes in the magneto-optical response were observed for the Ho, Yb, and Lu ion substitutions [89].

For example, different Gd^3+^ doping concentrations in the ferrite structure led to noticeable variations in the magnetic saturation values. This can be explained by the finite size effects of nanoparticles, indirect interactions of Fe-Gd and Co-Gd and relatively weaker Fe-Fe interactions, or surface dipole disorder induced by lattice distortions upon dopant substitution with different ionic radii [90]. These interactions can significantly influence the electromagnetic properties of spinel ferrites [91]. A low-density polyethylene/MWCNT/graphene/LaFe_2_O_3_ composite and La^3+^ ions substituted into CoLa_x_Fe_2−x_O_4_ and NiLa_x_Fe_2−x_O_4_ resulted in enhanced microwave absorption at an X-band frequency range of 8–12 GHz [92,93,94].

The nanocomposite Gd-doped MoS_2_/reduced graphene oxide Gd-MoS_2_/(rGO) for high-performance EMI shielding applications was prepared using a hydrothermal method with a varying percentage of Gd doping. The MoS_2_/rGO nanocomposite without dopant showed a low total SE, ~16.18 dB, and the one with 20% Gd-doped MoS_2_/rGO nanocomposite showed a higher total SE, ~20.47 dB, in the frequency range of 8.0–12.0 GHz due to enhanced electrical conductivity, defect dipole polarization, and interfacial polarization [95]. A 5% Gd-doped MoS_2_ electrode compound synthesized using cost-effective one-step hydrothermal methods can find applications in high-performance energy storage systems [96]. The importance and application of MoS_2_-based microwave-absorbing materials are in current social needs, including military radar stealth and civil electronic communication [97].

Flower-shaped Gd-doped FeNi_3_ and a novel raspberry-like absorbent based on a biomimetic design with high electromagnetic wave (EMW) absorption performance for an ultra-wide bandwidth of 12.24 GHz were designed by thermal catalysis [98]. These materials were investigated in the 2–18 GHz frequency range, while EMW attenuation was assigned to dipole polarization, multiple wave scattering, and reflections. According to free electron theory, closely related to electrical conductivity, the hierarchical flower-like and NiO/rGO composites were designed using the hydrothermal method. The NiO/rGO composites with a thickness of 2 mm showed an improved reflection loss of −60 dB at 9.8 GHz in comparison with a reflection loss of −40 dB at 10.8 GHz for NiO [99]. On the other hand, Pr^3+^-doped Co_0.5_Zn_0.5_Bi_0.4−x_Pr_0.1_Fe_1.5+x_O_4_ spinel ferrite series with different contents of Bi synthesized by the micro-emulsion method and Pr-doped (Bi_0.5_Na_0.5_)_1−x_Pr_x_TiO_3_ ceramics obtained by using the conventional solid-state method have remarkable potential for application in EMI shielding in the X-band frequency range of 8.2–12.4 GHz [100,101].

Neodymium (Nd)-doped spinel ferrites induce an increase in the polarization intensity, improving their saturation magnetization, electrical resistivity, thermal stability, and ability to absorb microwave radiation, making them suitable for advanced electronics, telecommunications, and power systems [102,103,104,105]. Classes of Ce-doped W-type barium ferrites, Ba_1−x_Ce_x_Ni_2_Fe_15.4_O_27_, with varying concentrations of Ce were successfully prepared by a sol–gel approach, whereby a Ce mole fraction of 0.3 gave the minimum reflection loss (RL) value of −52 dB, while a ferrite with Ce mole fraction of 0.4 had an RL value of −38 dB at 1.36 GHz frequency, suggesting the Ce enhancing EMW absorption of ferrites [106].

RE-metal-based MOFs have large specific surface areas. Their composites exhibit good dielectric loss, making them efficient EMW absorbers. The minimum RL value of the Ce(IV)-MOF is −32.12 dB at a frequency of 4.56 GHz at a thickness of 5.5 mm [107,108]. Excellent EMW absorption performance and mechanisms have been unveiled for the first time for Gd_2_O_2_S/rGO composites with an absorption capacity of −65 dB and an absorption bandwidth of 5.6 GHz. Gd_2_O_2_S nanosheets with a 1 nm thickness were produced via a facile hot injection method and mixed with rGO [109]. The EMW absorption performances (minimum reflection loss, RL, effective absorption bandwidth, **EAB**, thickness, and frequency range used) of various RE–ferrite composites, RE–transition metal intermetallics, REO composites, RE-MOFs, and other RE compounds are presented in [64].

#### 2.3.1. Synthesis, Morphology, and EMI Shielding Efficiency of RE-Based Materials

##### Synthesis of Gd_x_Fe_3−x_O_4_, x = 0, 0.02, 0.04, 0.06, 0.08, and 0.1

A series of Gd_x_F_e3−x_O_4_ materials with different Gd contents, x = 0, 0.02, 0.04, 0.06, 0.08, and 0.1, was obtained by using the hydrothermal method at a temperature of T = 180 °C and high vapor pressure. Gd(NO_3_)·6H_2_O, FeCl_3_·6H_2_O, and FeCl_2_·4H_2_O, as precursors of Gd^3+^, Fe^3+^, and Fe^2+^ ions, were dissolved in water separately and then mixed in the corresponding ratio, and the pH was adjusted up to the value of 12.8 using 10 M NaOH. The mixture was transferred to Teflon-lined stainless steel sealed containers and heated for 2 h at a temperature of 180 °C. After cooling, the obtained samples were washed several times with deionized water. For specific absorption rate (SAR) measurement, the obtained samples were functionalized by stirring overnight in a PEG solution [81].

##### Synthesis of CoFe_2−x_Dy_x_O_4_ Nanoparticles

**Figure 3:**
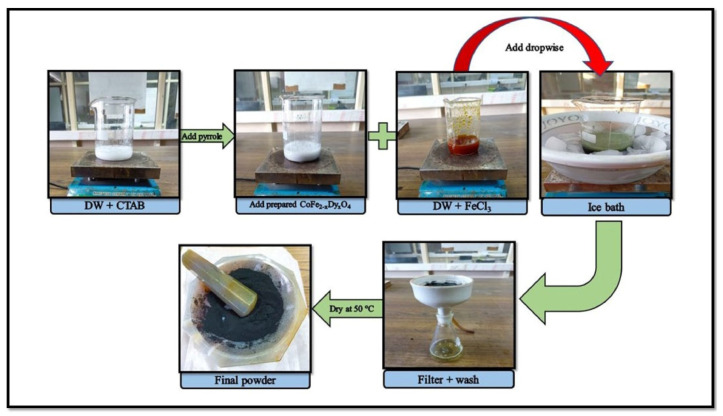
Schematic representation of in situ chemical oxidative polymerization technique. The figure is adapted from reference [110]. Copyright 2025, Elsevier B.V.

The first series of CoFe_2−x_Dy_x_O_4_ nanoparticles with a stoichiometric ratio of Co(NO_3_)_2_·6H_2_O, Fe(NO_3_)_3_·9H_2_O, and Dy(NO_3_)_3_·H_2_O precursor solutions in distilled water was synthesized by the co-precipitation approach. Oleic acid, followed by a liquid ammonia solution, was added dropwise at pH values of between 10 and 12. The obtained precipitates were washed and sintered at 500 °C for 2 h to form a fine powder. The nanocomposites of polypyrrole and 10 wt% CoFe_2−x_Dy_x_O_4_ were obtained via in situ chemical oxidative polymerization, and the synthesis is presented in detail in Figure 3 [110]. The obtained nanocomposites are promising candidates for applications in the 12.4 GHz to 18 GHz range, particularly in satellite communication systems, due to their impressive shielding performance, with an SE of approximately 16.3 dB at a shield thickness of 1.5 mm.

##### Synthesis of Gd- and Er-Doped α-MnO_2_ Nanorods

**Figure 4:**
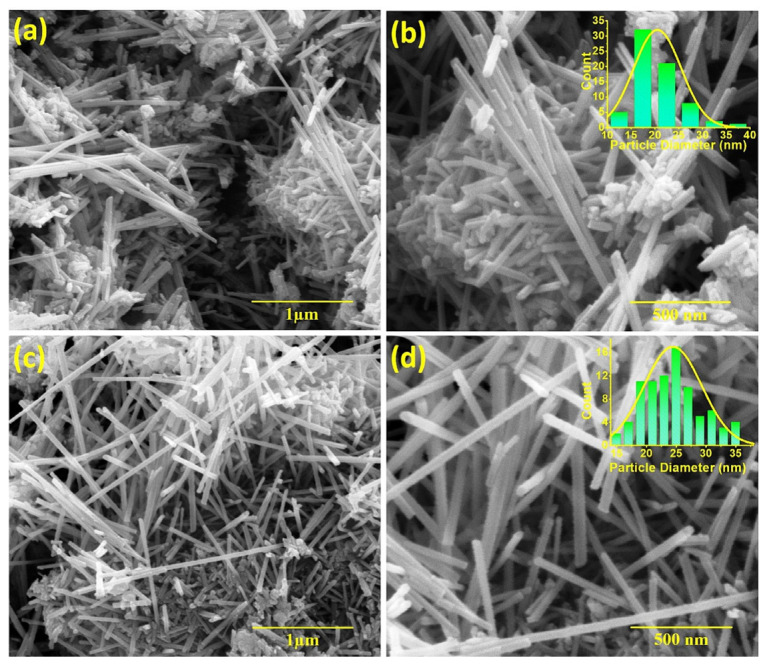
Morphology and particle size analysis by FESEM images at different resolutions of (**a**,**b**) Gd- and (**c**,**d**) Er-doped α-MnO_2_ samples. The figure is adapted from reference [111]. Copyright 2023, Elsevier B.V.

**Figure 5:**
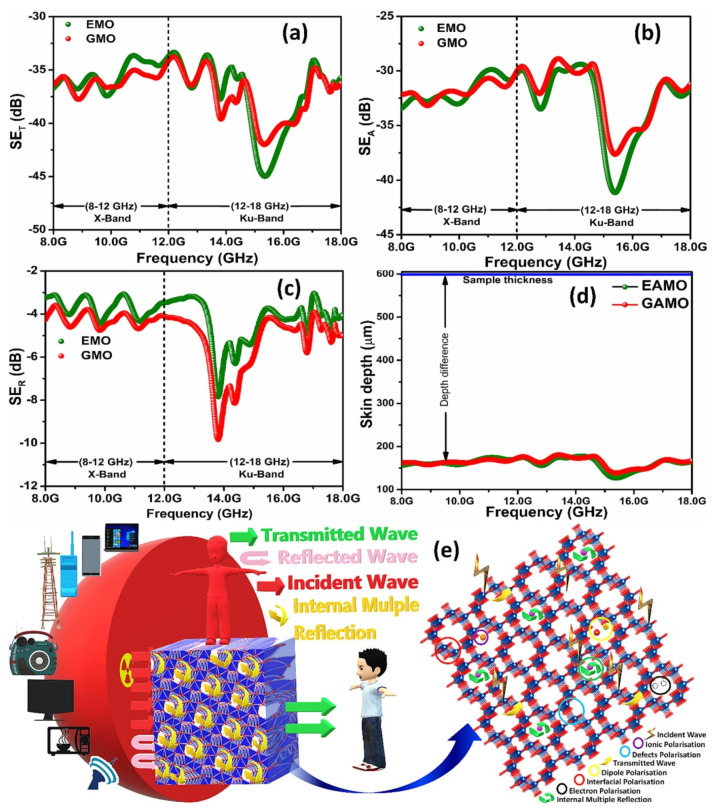
The EMI shielding efficiency of the Er-doped and Gd-doped α-MnO_2_ samples according to the (**a**) total, (**b**) absorption, (**c**) reflection, and (**d**) skin depth variation in the frequency range of 8 to 18 GHz. Schematic representation of (**e**) impact of hazardous EM waves radiated from various electronic sources on livelihood and demonstration of most possible EMI shielding mechanism to prevent it. The figure is adapted from reference [111]. Copyright 2023, Elsevier B.V.

Gd- and Er-doped α-MnO_2_ with a homogenously distributed rod-shaped morphology, as can be seen in Figure 4, was prepared by a modified chemical-route-assisted hydrothermal method. Er(CH_3_COO)_2_, Gd(CH_3_COO)_2_, and Mn(CH_3_COO)_2_, as precursors of Er, Gd, and Mn ions, were dissolved in deionized water separately, according to the stoichiometry ratio of 7 wt.% to PVA; KMnO_4_ was used as a reaction catalyst. The PVA was dissolved in hot (T = 80 °C), double-distilled water with continuous stirring. When it was dissolved in water, it became transparent. When the PVA became a transparent solution and cooled down to room temperature, the solution of Er/Gd and Mn ions precursors was added to it very slowly with constant stirring, followed by the addition of KMnO_4_. The pH value was adjusted to ~10 by adding KOH solution drop by drop, and the mixture of solutions was left for aging. The brownish-black precipitate was centrifuged and washed several times with distilled water, put into the hydrothermal reactor, and heated at 160 °C for 8 h. The brownish-black precipitate was again centrifuged, washed, dried additionally at 80 °C for 18 h, mortared, and annealed at 450 °C for 2 h to acquire a fine powder of Gd- and Er-doped α-MnO_2_ samples with good crystallinity. The EMI SE (presented in Figure 5a–d, showing total absorption and reflection and skin depth variation in the frequency range of 8 to 18 GHz) achieved a maximum of −43 dB at 15.3 GHz and −46 dB at 15.3 GHz for thin-layer Gd- and Er-doped α-MnO_2_ at a of thickness ~600 μm, respectively. A schematic representation of the impact of hazardous EM waves radiated from various electronic sources on livelihood and a demonstration of the most possible EMI shielding mechanism to prevent it are presented in Figure 5e [111].

Rare earth elements exhibit high magneto-dielectric effects, and their dielectric properties can be tuned by applying an electric or magnetic field. This property makes them attractive for use in electromagnetic shielding applications where the magnetic field can be used to control the dielectric constant of the material. For example, the doping of MnO_2_ with Er and Gd can enhance its dielectric properties, which make it a promising material for high-performance electronic devices. The incorporation of Er ions into the MnO_2_ matrix can potentially modify the material’s electrical conductivity, magnetic properties, and structural stability, thereby influencing its effectiveness in attenuating electromagnetic waves [111].

### 2.4. Iron Oxide-Based Materials as EMI Shielding Material

Iron oxides are inorganic compounds of iron and oxygen that exhibit very different stoichiometries due to the difference in the Fe oxidation state. Briefly, they can be classified into three groups: oxides of Fe^2+^ (FeO, wüstite), oxides of Fe^3+^ (various forms of Fe_2_O_3_, hematite, maghemite), and combined Fe^2+^/Fe^3+^ oxides (Fe_3_O_4_, magnetite, Fe_4_O_5_, Fe_5_O_6_, etc.). Apart from the difference in the Fe oxidation state, many existing crystal structures also enhance the diversity of iron oxides depending on the synthetic routes. For example, a variety of polymorphs of iron (III) oxide has been reported to date [112]. α-Fe_2_O_3_ is the most common form of iron (III) oxide and occurs as the mineral hematite. It has a rhombohedral structure and shows antiferromagnetic behavior below ~260 K and weak ferromagnetism at temperatures of between 260 and 950 K [113]. Common methods to prepare α-Fe_2_O_3_ are thermal decomposition and precipitation in the liquid phase. The β-phase of Fe_2_O_3_ is metastable, and at temperatures above 773 K, it converts to the α-phase. It can be obtained by the thermal decomposition of iron (III) salts. Similar to the β-phase, γ-Fe_2_O_3_ is also metastable and converts to the α-phase at high temperatures [114]. It has a cubic structure, and in the bulk form, it is ferromagnetic, but γ-Fe_2_O_3_ nanoparticles less than 10 nm in size show superparamagnetic behavior. The methods of γ-Fe_2_O_3_ preparation involve the thermal dehydration of gamma iron (III) oxide-hydroxide, the modest oxidation of _Fe3O4_, or the thermal decomposition of suitable iron salts [115]. The ε-phase is rhombic with properties between those of the alpha and gamma phases. It is metastable with a tendency to transform to the alpha phase at temperatures of between 773 and 1000 K. The synthesis of pure ε-Fe_2_O_3_ is challenging and involves the oxidation of iron in an electric arc or sol–gel precipitation from iron (III) nitrate [116]. In addition, an amorphous form of Fe_2_O_3_ also exists, which can be prepared under high pressure.

Oxides of iron have become interesting candidates for electromagnetic interference (EMI) shielding due to several factors. Fe_2_O_3_ is a magnetic material, while Fe_3_O_4_ is a ferromagnetic. Magnetic materials can interact with and absorb electromagnetic waves, especially at lower frequencies (like radio waves), thus reducing EMI. Iron oxides exhibit a certain level of electrical conductivity, allowing them to contribute to EMI shielding by reflecting or absorbing the electrical components of electromagnetic waves. In addition, they can contribute to the absorption of electromagnetic energy through dielectric loss, a mechanism that involves converting electromagnetic energy into heat. This makes it effective in dissipating energy and preventing the transmission of EMI. Finally, iron oxides are abundant, inexpensive, and easy to combine in composite materials, such as polymers or carbon-based materials, and can maintain their properties over time.

For EMI shielding applications, iron oxides are often combined with other materials, such as various polymers, graphene oxide, carbon nanotubes, or similar, to achieve desirable EMI shielding, mechanical strength, and electrical and thermal conductivity. Using an in situ polymerization technique, Azadmanjiri et al. created iron oxide and polypyrrole nanocomposites and examined their EMI shielding properties in the 0.1–18 GHz frequency range [117]. The composites’ intimate contact between the conducting and magnetic phases increased absorption by 10.10 dB at the instrument’s highest frequency limit (17–18 GHz), while iron oxide nanoparticles only increased the absorption by 2.6 dB. As the possible cause of this improvement, they proposed a better match between dielectric loss, magnetic loss, and improved dispersion of the magnetic/conductive nanocomposites in the matrix. Gupta et al. [118] reported that the microwave shielding properties were affected by the various morphologies of iron oxides. Using a two-step sol–gel process, they produced various ferrite structures, including cubes, rods, and flakes, covered with multilayer rGO. Their EMI shielding capabilities were evaluated in the Ku-band frequency range. In comparison to the flake- and cube-shaped iron oxides, the rod-shaped iron oxide covered with rGO sheets had the highest shielding efficiency value of ~33.30 dB (>99.9% attenuation). This resulted from the combined effect of magnetic and dielectric losses. Anisotropy energy in the composites, eddy current effects, and natural resonances were the sources of magnetic loss. The nanoferrite particle content of the composite was the main cause of eddy currents in the microwave ranges. The surface anisotropic field would result in higher anisotropy energy for the small materials due to the small-size effect. The higher anisotropy energy also contributed to greater microwave absorption. Moreover, the magnetic iron oxide was coated with an rGO layer, which increased the interfaces and surface polarization charges. Interfacial polarization is a significant polarization process, and the corresponding relaxation will lead to a loss mechanism. One of the main causes of dielectric loss could be interfacial polarization. It is well known that some heating-induced absorption losses are caused by the interaction of surface-formed molecular dipoles with the microwave field.

Dhawan et al. created a conducting ferrimagnetic PANI nanocomposite implanted with titanium dioxide (70–90 nm) and γ-Fe_2_O_3_ (9–12 nm) nanoparticles using micro-emulsion polymerization [119]. They discovered that the high shielding effectiveness value of −45 dB owing to absorption (SE_A_) was caused by dielectric and magnetic losses that resulted from the combined action of γ-Fe_2_O_3_ and TiO_2_. In contrast, the SE_A_ of PANI-TiO_2_ was around 22.4 dB, but that of PANI-γ-Fe_2_O_3_ was approximately 8.8 dB. In another work, PANI tubes made of rGO coated with γ-Fe_2_O_3_ nanoparticles were synthesized and characterized by Singh et al. [120]. The intercalated iron oxide nanoparticles were produced by thermally breaking down ferric acetyl acetonate in a reducing atmosphere. The β-naphthalene sulphonic acid-induced oxidative polymerization of aniline, which produced the core–shell shape, was also used to enclose those nanoparticles. At a thickness of 2.5 mm, the presence of rGO-γ-Fe_2_O_3_ in the PANI core structures increased the composite’s interfacial polarization and effective anisotropy energy, which increased scattering and produced a high shielding efficiency of about 51 dB.

Iron oxides are frequently combined with different carbon nanomaterials. The composite material can effectively absorb, reflect, and dissipate electromagnetic radiation over a wider frequency range by fusing the electrical conductivity of carbon nanotubes with the magnetic qualities of iron oxides. Fe_3_O_4_-nanoparticle-loaded functionalized multi-walled carbon nanotubes were produced by Bhaskara Rao et al. [121]. They discovered a high overall specific shielding efficiency of around 49.56 dB/(g cm^−3^), along with improved absorption (15.85 dB) and reflection (9.43 dB). Liu et al. [122] developed trilayer-type laminated nanocomposites with a matching layer of 15 wt.% Fe_3_O_4_, an absorbent layer of 5 wt.% MWCNTs, and a reflecting layer of 10 wt.% MWCNTs. Their results showed that such trilayer-type laminated nanocomposites have an excellent ability to absorb microwaves up to 40 dB in the 13 GHz to 40 GHz frequency range. Ferroferric oxide (Fe_3_O_4_) and MWCNTs were integrated by Li et al. into a core–shell system made of high-density polyethylene (HDPE), polyvinylidene fluoride (PVDF), and polystyrene (PS) [123]. The composite with MWCNTs in the PS shell and Fe_3_O_4_ in the PVDF matrix had the highest SE, measuring 25 dB at 9.5 GHz with 1 vol.% Fe_3_O_4_ and 1 vol.% MWCNTs. The SE was over 20 dB throughout the tested frequency range (X-band). Prasad et al. [124] developed a facile two-step hydrothermal process for the synthesis of a MoS_2_–reduced graphene oxide/Fe_3_O_4_ (MoS_2_-rGO/Fe_3_O_4_) nanocomposite and its application as an enhanced shielding material against electromagnetic interference. The Fe_3_O_4_ nanoparticles were spherical and evenly distributed throughout the MoS_2_-rGO composite. The MoS_2_-rGO/Fe_3_O_4_ nanocomposite was found to be an extremely effective electromagnetic shielding material in the 8.0–12.0 GHz X-band, according to an examination of its electromagnetic shielding efficiency. The MoS_2_-rGO composite showed low shielding performance (SE_T_ ~3.81 dB) compared to the MoS_2_-rGO/Fe_3_O_4_ nanocomposite (SE_T_ ~8.27 dB). This was caused by interfacial polarization in the presence of an electromagnetic field.

By the chemical oxidative polymerization of pyrrole, Sambyal et al. created a conducting polymer-based composite encapsulated with barium strontium titanate (BST), rGO, and Fe_3_O_4_ nanoparticles [125]. Filler components in the conducting polymer matrix produced an absorption-dominated shielding efficiency value of 48 dB in the 8.2–12.4 GHz (X-band) frequency range. Furthermore, the chemical and thermal stability of the composite material was enhanced by the use of magnetic and dielectric fillers. By using a self-assembly process, rGO was also added to latex coupled with magnetic iron oxide (Fe_3_O_4_) and flexible natural rubber [126]. Compared to NRG composites, Fe_3_O_4_ enhanced the electromagnetic interference shielding effectiveness (EMI SE) of natural rubber/reduced graphene oxide (NRG) composites. The EMI SE value of the NRMG composite with 10 parts per 100 parts of rubber rGO is 1.4 times higher than that of the NRG composite with the same rGO content in the 8.2–12.4 GHz frequency band. With a specific EMI SE of 26.4 dB mm^−1^, the NRMG composite outperforms the previously reported polymer/Fe_3_O_4_@rGO composites with a low rGO concentration. Remarkably, the NRMG composite’s EMI SE only decreases by 3.5% after 2000 bending–release cycles, indicating that it may find application in flexible shielding materials.

In order to create 3D network porous graphene nanoplatelet (GNP)/Fe_3_O_4_/epoxy nanocomposites with a low density of 0.34–0.73 g/cm^3^, Liu et al. suggested a new and simple method called epoxy–water–inorganic filler suspended emulsion polymerization [127]. The porous nanocomposite that resulted from loading 7 wt.% graphene nanoplatelets and 7 wt.% Fe_3_O_4_ nanoparticles showed a satisfactory specific electromagnetic interference (EMI) shielding effectiveness of about 37.03 dB/(g/cm^3^), which was much higher than that of the solid equivalents (28.30 dB/(g/cm^3^)). In another study, Fe_3_O_4_/thermally annealed graphene aerogel (Fe_3_O_4_/TAGA) was created by first thermally annealing ethylenediamine-functionalized Fe_3_O_4_ (NH_2_-Fe_3_O_4_) nanoparticles with graphene oxide (GO), followed by the addition of l-ascorbic acid [128]. Then, the Fe_3_O_4_/TAGA/epoxy nanocomposites were created using a template-casting technique. The resulting Fe_3_O_4_/TAGA/epoxy nanocomposites achieved the highest electromagnetic interference shielding effectiveness (EMI SE of 35 dB in the X-band) when the mass ratio of GO to NH_2_-Fe_3_O_4_ was 2:1 and the total mass fraction of Fe_3_O_4_/TAGA was 2.7 wt.% (comprising 1.5/1.2 wt.% Fe_3_O_4_/TAGA). This was significantly better than epoxy nanocomposites with the same Fe_3_O_4_/thermally annealed graphene oxide (Fe_3_O_4_/TAGO) loading, which only showed an EMI SE of 10 dB.

Liu et al. created magnetic reduced graphene oxide rGO@Fe_3_O_4_ nanoplatelets (NPs), which were used as fillers, by co-precipitation and electrostatic self-assembly [129]. The nanocomposites were made by applying external magnetic fields to align rGO@Fe_3_O_4_ NPs during epoxy curing. Because of the anisotropic properties of rGO@Fe_3_O_4_ NPs and external magnetic fields, the nanocomposite containing aligned rGO@Fe_3_O_4_ NPs showed anisotropic thermal conductivity. The produced sample exhibited exceptional thermal stability and 13.45 dB EMI shielding at 8.2 GHz. Overall, the rGO@Fe_3_O_4_ NPs’ in-plane interaction was enhanced by aligning them under a magnetic field, which promoted the growth of horizontal thermal conductive networks. Reflection is the main EMI shielding mechanism. Three-dimensional Fe_3_O_4_-decorated carbon nanotube/reduced graphene oxide foam/epoxy (3D Fe_3_O_4_-CNT/rGF/EP) nanocomposites with highly aligned three-dimensional structures were created by Liang et al. using a simple template approach [130]. The obtained 3D Fe_3_O_4_-CNT/rGF/EP nanocomposites with 0.24 wt.% rGF and 2.76 wt.% Fe_3_O_4_-CNTs showed a remarkable electrical conductivity of 15.3 S/m and an EMI SE of 36 dB within the X-band range, which was a nearly 482% improvement when compared to the EMI SE value of physically blended Fe_3_O_4_-CNT/EP nanocomposites without a three-dimensional structure (~6 dB). Using a supercritical carbon dioxide (Sc-CO_2_) foaming technique, lightweight and flexible methyl vinyl silicone rubber (VMQ)/multi-walled carbon nanotube (MWCNT)/ferriferrous oxide (Fe_3_O_4_) nanocomposite foams with superior EMI shielding capabilities were created [131]. The addition of a cellular structure and magnetic Fe_3_O_4_ nanoparticles greatly improved the VMQ/MWCNT/Fe_3_O_4_ foams’ microwave-absorbing capacity by successfully lowering secondary electromagnetic wave interference brought on by reflection. In the 8.2–12.4 GHz frequency range, these nanocomposite foams, which have an approximate density of 0.48 g/cm^3^, showed an EMI shielding efficiency (SE) of 27.5 dB and an average absorption ratio of up to 64%. The foams had a specific EMI SE of about 72 dB g^−1^ cm^3^ and a high conductivity of about 14.6 S/m with a filler loading of 1.78 vol.%.

Shu et al. [132] used rice-husk-based activated carbon (AC) and produced compo-sites with acicular or octahedral Fe_3_O_4_ nanoparticles. A significant effect on the EMI shielding effectiveness of the composites was assigned to the morphology of the metallic nanoparticles and the layered structure of the C component of the composites. They achieved an EMI SE of −52.14 dB.

Record values of EMI shielding effectiveness were achieved for MXene by incorporating magnetic nanoparticles as intercalators between layers [133] and 3D N-doped GO with silver nanowires (−79.99 dB) at a thickness of 2.660 mm [134].

## 3. Conclusions

This review has provided a comprehensive overview of the developments, classification, trends, and advances in new electromagnetic shielding materials. It gives a thorough literature review of the EMI shielding properties of new EMI shielding materials such as biochars, scaffolds, rare earth, and ferrite-based materials. A detailed discussion is given regarding the preparation methods, structure, EMI shielding performance, EMI shielding mechanisms, and application perspectives of these materials.

Looking ahead, the prospects for and research on electromagnetic shielding materials should focus on scalability, improving performance, cost-effectiveness, sustainability, versatility, and production methods to advance new materials like biochars, scaffolds, rare earth, and ferrite-based materials as novel EMI shielding solutions. Biochars are promising eco-friendly, sustainable, and renewable materials that can be potentially used as EMI shielding materials in electronic devices and construction materials, similar to scaffolds, new-generation, easy-to-manufacture materials with excellent EMI shielding performance. Although MXene, carbon nanotubes, and graphene-based composites are highly efficient EMI shielding materials due to their large free surface area, high aspect ratio, chemical stability, and lightweight nature, the large number of synthetic phases leads to their high price. Carbon-based nanomaterials derived from biomass are being developed in a quest for new, cost-efficient, sustainable, and affordable materials. 

The current disadvantages of these materials are the thickness of the shielding barrier and lack of transparency, which limit some applications.

Materials doped with rare earth elements have been developing over the last few years. They have specific structures, interfacial polarization, multiple reflections, and excellent conductive and EMI shielding properties. Combining the magnetic properties of ferrite-based materials with the electrical conductivity of carbon nanomaterials, composite materials can efficiently absorb, reflect, and dissipate electromagnetic energy across a broader frequency range. Although the materials showed outstanding EMI shielding performance, the price of REEs will limit the application of these composites to more sophisticated and sensitive fields such as medicine.

Their EMI shielding effectiveness, good magnetic and dielectric properties, excellent thermal stability, and high electrical conductivity and mechanical strength make biochars, scaffolds, rare earth, and ferrite-based materials ecological and sustainable solutions as new products for blocking EMWs.

## Figures and Tables

**Table 1:** Summarized results for biochar and scaffolds as EMI shielding materials.

Material	Reinforcement and Matrix Thickness	EMI Shielding Effectiveness	Frequency	
Bamboo charcoal/HMWPE/LLDPE composite	3 mm, 140 mm	48.7 dB	1500 MHz	[39]
Pine chip/PLA composite	0.25 mm	>32 dB	18–26.5 GHz	[4]
MXene aerogel/WPC composite	3 mm	71.3 dB	8.5–12.5 GHz	[1]
Gypsum–biochar drywall-like composite	>2 mm	11.65 ± 1.6 dB, 19.2 ± 5.7 dB, and 19.25 ± 1.8 dB for 10, 20, and 40% *w*/*w* biochar contents	6 GHz	[43]
Drywall panels coated with commercial wood biochar	10 mm	17 dB 25 dB	1 GHz 18 GHz	[47]
Cement-based/commercial lignin-derived biochar/PVC composite	4 mm	16 dB	5.4–8 GHz	[48]
EBC@CNF@MWCNT composite	~8 mm	>32 dB	8–12 GHz	[36]
Olive tree-derived biochar/polytetrafluoroethylene composite	0.1–0.5 mm	39 dB	1–3 GHz	[49]
Apple and quince biowaste-based biochars	0.2 mm	15.5 dB	8–12 GHz	[50]
*Ricinus communis* outer shell-based biochar/epoxy composite	0.15 mm	26.5 dB	8–12 GHz	[37]
18 wt.% lignin-based biochar/cementitious composite	4 mm	15 dB	10 GHz	[57]
Cashew shell biochar/carbon fiber-reinforced epoxy resin composite	not reported	−48.6 dB	18 GHz	[58]
Jackfruit rag biochar/waste silk fiber-reinforced vinyl ester composite	not reported	31.5 dB 47.25 dB 63 dB 68.25 dB	8 GHz 12 GHz 16 GHz 18 GHz	[59]
Cellulose scaffold/AgNP composite	1 mm	69.1 dB	8.2–12.4 GHz	[52]
Carbon scaffold/polyurethane/Fe_3_O_4_ NP composite	~8 mm	32 dB	8.2–12.4 GHz	[53]
Cellulose-paper-based scaffold/Fe_3_O_4_ NP composite	1.3 mm	1805.9 dB/cm^2^ g	10.3 GHz	[54]
3D cellulose scaffold/CNT/MXene composite	0.25 mm	29.3 dB	18–26.5 GHz	[55]
Hybrid scaffold coupled with high-quality graphene array/MXene-Co aerogel	6 mm	72.86 dB	8.2–12.4 GHz	[56]
3D-printed scaffold/CNTs	10 mm	35.9 dB	8.2–12.4 GHz	[51]

## Data Availability

This review article does not contain any original data. All data referenced in this article are publicly available from the sources cited in the references. No new datasets were generated or analyzed in this work.

## References

[1] Liang C., Qiu H., Song P., Shi X., Kong J., Gu J. (2020). Ultra-light MXene aerogel/wood-derived porous carbon composites with wall-like “mortar/brick” structures for electromagnetic interference shielding. Sci. Bull..

[2] Li J., Cui M., Wen J., Chen Y., Shi B., Fan H., Xiang J. (2021). Leather-like hierarchical porous composites with outstanding electromagnetic interference shielding effectiveness and durability. Compos. B Eng..

[3] Manassas A., Apostolidis C., Iakovidis S., Babas D., Samaras T. (2023). A study of the long term changes in the electromagnetic environment using data from continuous monitoring sensors in Greece. Sci. Rep..

[4] Tolvanen J., Hannu J., Hietala M., Kordas K., Jantunen H. (2019). Biodegradable multiphase poly(lactic acid)/biochar/graphite composites for electromagnetic interference shielding. Compos. Sci. Technol..

[5] Calvente I., Núñez M.I. (2024). Is the sustainability of exposure to non-ionizing electromagnetic radiation possible?. Med. Clín..

[6] Bandara P., Carpenter D.O. (2018). Planetary electromagnetic pollution: It is time to assess its impact. Lancet Planet. Health.

[7] Karimi A., Ghadiri Moghaddam F., Valipour M. (2020). Insights in the biology of extremely low-frequency magnetic fields exposure on human health. Mol. Biol. Rep..

[8] Lin Y., Gao P., Guo Y., Chen Q., Lang H., Guo Q., Miao X., Li J., Zeng L., Guo G. (2021). Effects of long-term exposure to L-band high-power microwave on the brain function of male mice. Biomed Res. Int..

[9] Touitou Y., Selmaoui B., Lambrozo J. (2022). Assessment of cortisol secretory pattern in workers chronically exposed to ELF-EMF generated by high voltage transmission lines and substations. Environ. Int..

[10] Alshammary R.N., Mohammed Zaki Z.D., Al-Haaik A.G. (2022). Effect of mobile frequencies exposure on histology of retina and cornea in pregnant albino mice. Iraqi J. Vet. Sci..

[11] Tuhanioğlu B., Erkan S.O., Gürgen S.G., Özdaş T., Görgülü O., Çiçek F., Günay İ. (2019). The effect of very low dose pulsed magnetic waves on cochlea. Braz. J. Otorhinolaryngol..

[12] Dunham A., Pegg J.R., Carolsfeld W., Davies S., Murfitt I., Boutillier J. (2015). Effects of submarine power transmission cables on a glass sponge reef and associated megafaunal community. Mar. Environ. Res..

[13] Harsanyi P., Scott K., Easton B.A.A., de la Cruz Ortiz G., Chapman E.C.N., Piper A.J.R., Rochas C.M.V., Lyndon A.R. (2022). The effects of anthropogenic electromagnetic fields (EMF) on the early development of two commercially important crustaceans, *European lobster*, *Homarus gammarus* (L.) and *Edible crab*, *Cancer pagurus* (L.). J. Mar. Sci. Eng..

[14] Piccinetti C.C., De Leo A., Cosoli G., Scalise L., Randazzo B., Cerri G., Olivotto I. (2018). Measurement of the 100 MHz EMF radiation in vivo effects on zebrafish D. rerio embryonic development: A multidisciplinary study. Ecotoxicol. Environ. Saf..

[15] Boga A., Emre M., Sertdemir Y., Uncu İ., Binokay S., Demirhan O. (2016). Effects of GSM-like radiofrequency irradiation during the oogenesis and spermiogenesis of *Xenopus laevis*. Ecotoxicol. Environ. Saf..

[16] Chen L., Mai T., Ji X.-X., Wang P.-L., Qi M.-Y., Liu Q., Ding Y., Ma M.-G. (2023). 3D printing of customizable and lightweight multilayer MXene/nanocellulose architectures for tunable electromagnetic interference shielding via direct ink writing. Chem. Eng. J..

[17] Chung D.D.L. (2020). Materials for electromagnetic interference shielding. Mater. Chem. Phys..

[18] Orasugh J.T., Ray S.S. (2023). Functional and structural facts of effecttive electromagnetic interference shielding materials: A review. ACS Omega.

[19] Li Y., Wu H., Deng K., Jiang J., Yang Z., Zhang R. (2023). Preparation of graphite/ferrite/resin-based composite high-efficiency wave-absorbing materials by selective laser sintering. Adv. Eng. Mater..

[20] Ji Z., Wang Q., Wang Z., Duan Y., Dong C., Liaw P.K. (2022). Electromagnetic wave-absorbing behavior of soft-magnetic medium entropy alloys with BCC/L21 coherent microstructure. Mater. Des..

[21] Zhao Y., Lin Z., Huang L., Meng Z., Yu H., Kou X., Zou Z., Huang P., Wang Y., Xi D. (2023). Simultaneous optimization of conduction and polarization losses in CNT@NiCo compounds for superior electromagnetic wave absorption. J. Mater. Sci. Technol..

[22] Zhou Y., Zhu Y., Chen P., Li X. Effect of rare earth-transition metal electronic interaction on magnetism in FeCoEr alloys. J. Rare Earths.

[23] Liu H., Yang S., Wang G., Liu H., Peng Y., Sun C., Li J., Chen J. (2022). Strong electronic orbit coupling between cobalt and single-atom praseodymium for boosted nitrous oxide decomposition on Co_3_O_4_ catalyst. Environ. Sci. Technol..

[24] Wang H., Zhang H., Zhao K., Nie A., Alharthi S., Amin M.A., El-Bahy Z.M., Li H., Chen L., Xu B.B. (2023). Research progress on electromagnetic wave absorption based on magnetic metal oxides and their composites. Adv. Compos. Hybrid Mater..

[25] Jha A.R. (2014). Rare Earth Materials: Properties and Applications.

[26] Wen H., Zhao W., Han X. (2022). Constructing Co_3_O_4_/La_2_Ti_2_O_7_ p-n heterojunction for the enhancement of photocatalytic hydrogen evolution. Nanomaterials.

[27] Legvold S. (1980). Chapter 3: Rare earth metals and alloys in book. Handb. Ferromagn. Mater..

[28] Wang Y., Wu X., Zhang W., Chen W. (2016). Synthesis and electromagnetic properties of La-doped Ni–Zn ferrites. J. Magn. Magn. Mater..

[29] Kumar P., Pathak S., Singh A., Verma R., Khanduri H., Jain K., Tawale J., Wang L., Pant R.P. (2024). Augmented magnetic nanoparticle assimilation in rGO sheets for tailored static and dynamic magnetic properties in surface functionalized Co_0.8_Zn_0.2_Fe_2_O_4_ nanoferrite–rGO hybrid structures. J. Mater. Chem. C.

[30] Pathak S., Verma R., Singhal S., Chaturvedi R., Kumar P., Sharma P., Pant R.P., Wang X. (2021). Spin dynamics investigations of multifunctional ambient scalable Fe_3_O_4_ surface decorated ZnO magnetic nanocomposite using FMR. Sci. Rep..

[31] Ma Z., Jiang R., Jing J., Kang S., Ma L., Zhang K., Li J., Zhang Y., Qin J., Yun S. (2024). Lightweight Dual-Functional Segregated Nanocomposite Foams for Integrated Infrared Stealth and Absorption-Dominant Electromagnetic Interference Shielding. Nano-Micro Lett..

[32] Ma Y., Jiang R., Zhang Y., Ma L., Bai Y., Zhang K., Zuo X., Zuo Y., Jing H., Qin J. (2025). Lightweight and mechanically strong MXene-Based microcellular nanocomposite foams for integrated electromagnetic interference shielding and thermal management. Compos. Sci. Technol..

[33] Kruželák J., Kvasničáková A., Hložeková K., Dosoudil R., Gořalík M., Hudec I. (2021). Electromagnetic Interference Shielding and Physical-Mechanical Characteristics of Rubber Com-posites Filled with Manganese-Zinc Ferrite and Carbon Black. Polymers.

[34] Chu W., Li J., Lin J., Li W., Xin J., Liu F., He X., Ma Z., Zhao Q. (2024). Honeycomb-like Polyimide/Fe_3_O_4_@PPy foam for electromagnetic wave shielding with excellent absorption characteristics. Compos. Sci. Technol..

[35] Zhou Z., Song Q., Huang B., Feng S., Lu C. (2021). Facile Fabrication of Densely Packed Ti3C2 MXene/Nanocellulose Composite Films for Enhancing Electromagnetic Interference Shielding and Electro-/Photothermal Performance. ACS Nano.

[36] Miao Y., Lin J., Wang E., Liang Y., Li W., Dai C., Huang J., Zhang W. (2023). Electrically conductive bamboo charcoal@cellulose nanofibrils based composite membranes designed for electromagnetic interference shielding and flame retardant. Ind. Crop. Prod..

[37] Perumal R.S., Muralidharan B. (2024). Valorization of *Ricinus communis* outer shell biomass to biochar: Impact of thermal decomposition temperature on physicochemical properties and EMI shielding performance. Results Eng..

[38] Tomczyk A., Sokołowska Z., Boguta P. (2020). Biochar physicochemical properties: Pyrolysis temperature and feedstock kind effects. Rev. Environ. Sci. Biotechnol..

[39] Li S., Huang A., Chen Y.-J., Li D., Turng L.-S. (2018). Highly filled biochar/ultra-high molecular weight polyethylene/linear low density polyethylene composites for high-performance electromagnetic interference shielding. Compos. B Eng..

[40] Gabhi R.S., Kirk D.W., Jia C.Q. (2017). Preliminary investigation of electrical conductivity of monolithic biochar. Carbon.

[41] Gokce E.C., Calisir M.D., Selcuk S., Gungor M., Acma M.E. (2024). Electromagnetic interference shielding using biomass-derived carbon materials. Mater. Chem. Phys..

[42] Akgül G., Demir B., Gündoğdu A., Türk A.S., Sözer S. (2020). Biochar-iron composites as electromagnetic interference shielding material. Mater. Res. Express.

[43] Natalio F., Corrales T.P., Feldman Y., Lew B., Graber E.R. (2020). Sustainable lightweight biochar-based composites with electromagnetic shielding properties. ACS Omega.

[44] Savi P., Yasir M. (2020). Waveguide measurements of biochar derived from sewage sludge. Electron. Lett..

[45] Savi P., Yasir M., Bartoli M., Giorcelli M., Longo M. (2020). Electrical and Microwave Characterization of Thermal Annealed Sewage Sludge Derived Biochar Composites. Appl. Sci..

[46] Yasir M., Zaccagnini P., Palmara G., Frascella F., Paccotti N., Savi P. (2021). Morphological Characterization and Lumped Element Model of Graphene and Biochar Thick Films. C.

[47] Savi P., Ruscica G., di Summa D., Natali Sora I. (2022). Shielding effectiveness measurements of drywall panel coated with biochar layers. Electronics.

[48] Ruscica G., Peinetti F., Natali Sora I., Savi P. (2024). Analysis of electromagnetic shielding properties of cement-based composites with biochar and PVC as fillers. C.

[49] Nikolopoulos C.D., Baklezos A.T., Kapetanakis T.N., Vardiambasis I.O., Tsubota T., Kalderis D. (2023). Characterization of the electromagnetic shielding effectiveness of biochar-based materials. IEEE Access.

[50] Milenkovic M., Saeed W., Yasir M., Nassar K.E.S., Syrgiannis Z., Milivojevic D., Spanopoulos I., Azmy A., Bajuk-Bogdanovic D., Maletić S. (2024). Carbonized apples and quinces stillage for electromagnetic shielding. Nanomaterials.

[51] Wang C., Wang Y., Zou F., Fang B., Zhao J., Zhang H., Guo J., Jia L., Yan D. (2025). Construction of lightweight, high-energy absorption 3D-printed scaffold for electromagnetic interference shielding with low reflection. Compos. B Eng..

[52] Tran T.T.V., Vo D.-V.N., Nguyen S.T., Luu S.D.N., Mofijur M., Vu C.M. (2021). In situ sintered silver decorated 3D structure of cellulose scaffold for highly thermoconductive electromagnetic interference shielding epoxy nanocomposites. J. Appl. Polym. Sci..

[53] He H., Wang Y., Zhao Z., Wang Q., Wei Q., Cai Y. (2022). Dual-encapsulated multifunctional phase change composites based on biological porous carbon for efficient energy storage and conversion, thermal management, and electromagnetic interference shielding. Energy Storage Mater..

[54] Wei Z., Cheng Y., Hu X., Meng Y., Zhan Y., Li Y., Xia H., Jiang X., Chen Z. (2024). Cellulose–derived carbon scaffolds with bidirectional gradient Fe_3_O_4_ distribution: Integration of green EMI shielding and thermal management. Int. J. Biol. Macromol..

[55] Wang Z.-X., Han X.-S., Zhou Z.-J., Meng W.-Y., Han X.-W., Wang S.-J., Pu J.-W. (2021). Lightweight and elastic wood-derived composites for pressure sensing and electromagnetic interference shielding. Compos. Sci. Technol..

[56] Hu B., Guo H., Cui Y., Li J., Cao M., Qi W., Cao X., Li B. (2024). Engineering multifunctional phase change composites enabled by dual-interpenetrating hybrid scaffold for excellent thermal conductivity and electromagnetic absorption. J. Chem. Eng..

[57] Yasir M., di Summa D., Ruscica G., Natali Sora I., Savi P. (2020). Shielding properties of cement composites filled with commercial biochar. Electronics.

[58] Babu M., Bapu B.R.T., Muruganantham P., Anita R., Nagaraju V., P. J. Kumar S. (2023). Role of cashew shell biochar on EMI shielding behaviour of carbon fibre–epoxy nanocomposites in E, F, I and J band–microwave frequencies. Biomass Convers. Biorefinery.

[59] Suresh N., Sivakumar P., Malathi A.C.J., Balamurugan K.S. (2025). Effect of temperature on EMI shielding behavior of jack fruit rags biochar and waste silk fiber-reinforced vinyl ester composite. J. Mater. Sci. Mater. Electron..

[60] Sharma S., Parne S.R., Srihari S., Panda S., Gandi S. (2024). Progress in microwave absorbing materials: A critical review. Adv. Colloid Interface Sci..

[61] Nikzad A., Parvizi R. (2020). Presence of neodymium and gadolinium in cobalt ferrite lattice: Structural, magnetic and microwave features for electromagnetic wave absorbing. J. Rare Earth.

[62] Zhang C., Wang L., Ji X., Chen L., Yan H., Xing Z., Wang B. (2024). Preparation and electrorheological behavior of rare-earth La ion doping MIL-125 nanoparticles. Adv. Eng. Mater..

[63] Isari A.A., Ghaffarkhah A., Hashemi S.A., Wuttke S., Arjmand M. (2024). Structural design for EMI shielding: From underlying mechanisms to common pitfalls. Adv. Mater..

[64] Qiao J., Li L., Liu J., Wu N., Liu W., Wu F., Zeng Z. (2024). The vital application of rare earth for future high-performance electromagnetic wave absorption materials: A review. J. Mater. Sci. Technol..

[65] Deng Y., Wang L., Liu W., Wu N., Liu J., Pan F., Zeng Z. (2024). Research progress on controllable absorption properties of rare earth element doped electromagnetic wave absorbing materials. Chin. J. Chem..

[66] Mohapatra P.P., Ghosh S., Jain A., Aich S., Dobbidi P. (2023). Rare earth substituted lithium ferrite/carbon black ceramic composites for shielding electromagnetic radiation. J. Magn. Magn. Mater..

[67] Zhu X., Zhang J., Liu J., Zhang Y. (2019). Recent progress of rare-earth doped upconversion nanoparticles: Synthesis, optimization, and applications. Adv. Sci..

[68] Liu J., Pan Y., Yu L., Gao Z., Zhang S., Lan D., Jia Z., Wu G. (2025). MoS2-based composites for microwave absorption mechanism-oriented structural optimization and design perspectives. Carbon.

[69] Prasad J., Singh A.K., Gahlot A.P.S., Tomar M., Gupta V., Singh K. (2021). Electromagnetic interference shielding properties of hierarchical core-shell palladium-doped MoS2/CNT nanohybrid materials. Ceram. Int..

[70] Zhang H., Ma G., Wu H., Yuan M., Liu X., Huang Z. (2024). Enhanced mechanical and wave-absorption properties of SiC–Si_3_N_4_–FeSi porous ceramics by introducing Al. J. Am. Ceram. Soc..

[71] Wang L., Chen S., Zhu X., Chen J., Liang J., Wang M. (2024). Influence of various rare earth elements co-integration on the properties of super multifunctional metal-based materials fabricated by laser cladding. Appl. Mater. Today.

[72] Li Z., Mi W., Bai H. (2018). The role of rare–earth dopants in tailoring the magnetism and magnetic anisotropy in Fe_4_N. J. Phys. Chem. Solids.

[73] Zhang W., Zhao B., Xiang H., Dai F.-Z., Wu S., Yanchun Z. (2021). One-step synthesis and electromagnetic absorption properties of high entropy rare earth hexaborides (HEREB_6_) and high entropy rare earth hexaborides/borates (HEREB_6_/HE REBO_3_) composite powders. J. Adv. Ceram..

[74] Chen H., Zhao B., Zhao Z., Xiang H., Dai F.-Z., Liu J., Zhou Y. (2020). Achieving strong microwave absorption capability and wide absorption bandwidth through a combination of high entropy rare earth silicide carbides/rare earth oxides. J. Mater. Sci. Technol..

[75] Zámborszky F., Gyüre Garami B., Jánosi B., Vajtai L., Hegyessy L., Gresits I., Simon F. (2022). High-frequency characterization of Fe-based nanocrystalline cores. J. Magn. Magn. Mater..

[76] Azmat M., Yang J., Li Q., Zhang J., Haibo J., Kashif N.M., Li J. (2024). Role of 4f electrons and 3d-4f hybridization in metal-insulator transition in RE (La, Nd, Sm, Eu, Dy and Er)-doped vanadium dioxide for thermochromic applications. Ceram. Int..

[77] Nagaraj N., Manjunatha H.C., Vidya Y.S., Seenappa L., Sridhar K.N., Gupta P.S.D. (2022). Investigations on lanthanide polymers for radiation shielding purpose. Radiat. Phys. Chem..

[78] Hasan M.S., Khan M.I., Mandal G., Awais M., Farhat L.B., Liu J. (2025). Integrating the structural, electro-optical, dielectric, and magnetic features of Co–Mg–La ferrites/graphene composites. J. Am. Ceram. Soc..

[79] Jia Z., Gao Z., Kou K., Feng A., Zhang C., Xu B., Wu G. (2020). Facile synthesis of hierarchical A-site cation deficiency perovskite La_x_FeO_3−y_/RGO for high efficiency microwave absorption. Compos. Commun..

[80] Serrano A., García-Martín E., Granados-Miralles C., Gorni G., López-Sánchez J., Ruiz-Gómez S., Pérez L., Quesada A., Fernández J.F. (2021). Hexaferrite-based permanent magnets with upper magnetic properties by cold sintering process via a non-aqueous solvent. Acta Mater..

[81] Kahil H., Faramawy A., El-Sayed H., Abdel-Sattar A. (2021). Magnetic properties and SAR for gadolinium-doped iron oxide nanoparticles prepared by hydrothermal method. Crystals.

[82] Choudhary N., Verma M.K., Sharma N.D., Sharma S., Singh D. (2020). Correlation between magnetic and transport properties of rare earth doped perovskite manganites La_0.6_R_0.1_Ca_0.3_MnO_3_ (R = La, Nd, Sm, Gd, and Dy) synthesized by Pechini process. Mater. Chem. Phys..

[83] http://abulafia.mt.ic.ac.uk/shannon/ptable.php.

[84] Rajeshwari A., Punithavathy I.K., Jeyakumar S.J., Jothibas M. (2023). Dependance of gadolinium ions on structural, magnetic and dielectric properties of manganese nanoferrites. Mater. Chem. Phys..

[85] Kadam A.B., Mande V.K., Kadam S.B., Kadam R.H., Shirsath S.E., Borade R.B. (2020). Influence of gadolinium (Gd^3+^) ion substitution on structural, magnetic and electrical properties of cobalt ferrites. J. Alloys Compd..

[86] Geetha P., Taddesse P., Murali N., Narayana P.V.L. (2022). Impact of Gd^3+^ and Nd^3+^ ions substitution on structural and magnetic properties of Co_0.5_Ni_0.5_Fe_2_O_4_ ferrite system. J. Indian Chem. Soc..

[87] Manner O., Sarmah S., Patra K.P., Maji D., Ravi S., Bora T. (2024). Effect of rare earth (Ho and Er) co-substitution on the magnetic and dielectric properties of nanocrystalline cobalt ferrites. Ceram. Int..

[88] Mohapatra P.P., Dobbidi P. (2023). Development of spinel ferrite-based composites for efficient EMI shielding. Mater. Chem. Phys..

[89] Cheng F., Jia J., Xu Z., Zhou B., Liao C., Yan C.-H., Chen L.-Y., Zhao H.-B. (1999). Microstructure, magnetic and magneto-optical properties of chemical synthe sized Co–RE (RE, Ho, Er, Tm, Yb, Lu) ferrite nanocrystalline films. J. Appl. Phys..

[90] Bansal M., Ahlawat S.D., Singh A., Rathee S.P., Kumar V., Maan A., Singh M. (2025). Sol-gel synthesized CoFe_2−x_Gd_x_O_4_:SiO_2_ nanocomposites for structural, thermal and magnetic investigation. Next Mater..

[91] Srinivas C., Praveen K.N., Kumar E.R., Singh S., Meena S.S., Bhatt P., Rao C.T.V., Sarkar D., Arun B., Raju K.C.J. (2022). Microwave absorption properties of rare earth (RE) ions doped Mn–Ni–Zn nanoferrites (RE = Dy, Sm, Ce, Er) to shield electromagnetic interference (EMI) in X-band frequency. Ceram. Int..

[92] Kumar T.M.M., Kini H.J., Praveen M., Kumar M. (2024). Electromagnetic interference shielding performance of lanthanum ferrite with MWCNT and graphene in the polyethylene polymer matrix in X-band frequency. Diam. Relat. Mater..

[93] Mashadi Y., Winatapura D.S., Setiawan J., Mulyawan A., Fakhrudin M., Taryana Y., Sudrajat N., Adi W.A., Gunanto Y.E. (2024). Microwave absorbing material of cobalt lanthanum ferrite: The contributions of intrinsic and extrinsic factors on the microwave absorption properties. Appl. Phys. A.

[94] Yunasfi, Dewi S.H., Mashadi, Winatapura D.S., Setiawan J., Mulyawan A., Edi Gunanto Y., Ari Adi W. (2024). Exploring the structural and magnetic properties of La-doped nickel ferrite for microwave absorbing application. J. Magn. Magn. Mater..

[95] Prasad J., Singh K.A., Haldar K.K., Gupta V., Singh K. (2019). Electromagnetic interference shielding effectiveness in 3D flower-like MoS_2_-rGO/gadolinium-doped nanocomposites. J. Alloys Compd..

[96] Das S., Banerjee A., Pal P., Rudra S., Nandi U., Ghosh A. (2024). Hydrothermally synthesized gadolinium doped molybdenum disulfide for electrochemical supercapacitor applications. J. Energy Storage.

[97] Li J., Li X., He L., Guo H., Xia W., Sun B., Cao C., Sha L., Zhou D. (2024). MoS_2_-based nanocomposites for microwave absorption: A review. ACS Appl. Nano Mater..

[98] Hu R., He X., Luo Y., Liu C., Liu S., Lv X., Yan J., Peng Y., Yuan M., Che R. Biomimetic multi-interface design of raspberry-like absorbent: Gd-doped FeNi_3_@covalent organic framework derivatives for efficient electromagnetic attenuation. Small Methods.

[99] Phani P.S.D.R., Sahu S., Gurrala R.C., Dobbidi P., Raidongia K., Latha B.S., Babu B.K., Annapurna N. (2025). Leveraging synergistic interfaces in NiO and NiO/rGO heterostructures for enhanced microwave absorption. Surf. Interface.

[100] Akbar R., Shifa M.S., Saleem A., Zaib A., Raheem F., Khaliq M.W. (2022). Tuning the properties of praseodymium cobalt-zinc ferrites by substitution of bismuth. J. Mat. Phy. Sci..

[101] Das D., Dikshit A.P., Samal R.R., Parashar K., Parashar S.K.S. (2025). A-site Pr-doped BNT ceramics for absorption-dominated EMI shielding in X-band. J. Mater. Sci. Mater. Electron..

[102] Seethalakshmi K., Sakthipandi K., Sethuraman B., Alhashmi B., Venkatesan K., Rajkumar G., Alqarni A.S., Ansari I.A., Raghavan M.S. (2025). Investigation of electromagnetic shielding effectiveness and magnetic phase transitions of neodymium-doped Cu_0.25_Ni_0.5_Zn_0.25_Fe_2−*x*_Nd*_x_*O_4_ nanoferrites. J. Rare Earth.

[103] Majeed M., Akhtar M., Khatoon R., Amin N., Morley N., Tung L.D., Amami M., Abbas W., Siddeeg S.M., Thanh N.T.K. (2024). Dielectric and magnetic response of Cu-Co-Sm ferrite impregnated with graphene nanoplatelets for high-frequency device applications. J. Alloys Compd..

[104] Kanna R.R., Jaisiva S., Dhineshbabu N.R., Kesavan M.P., Lenin N., Prasad L.G., Kumar P.R. (2024). Microwave-absorbing behavior of rare-earth-ion-doped copper manganese nanoferrites in X-band frequency. Ceram. Int..

[105] Kumar P.V.P., Suryanarayana B., Vemula V.L., Rao D.J., Uppugalla S., Ramakrishna Y. (2023). Effect of rare earth ions (RE = La^3+^, Sm^3+^, Nd^3+^, and Gd^3+^) substitution on structural, magnetic properties, and dc electrical resistivity of Co_0.5_Ni_0.5_Fe_2_O_4_ ferrite. Appl. Phys. A.

[106] Deng Y., Li L., Wang L., Wu N., Jin H., Gao F., Zeng Z. (2024). Rare earth Ce-doped W-type barium ferrites for tunable electromagnetic waves absorption performance. Mater. Res. Bull..

[107] Yang Y., Jin P., Li Y., Li S., Zhu L. (2024). The fabrication of Ce-MOFs with the effective electromagnetic wave absorption performance. Inorg. Chem. Commun..

[108] Deng Y., Guo H., Zhao C., Huang J., Song F., Zeng X. (2025). Rare-earth metal-based CuLa@NC nanorods anchoring Mo-MXene layers for electromagnetic wave absorption. Colloids Surf. A Physicochem. Eng. Asp..

[109] Li N., Wen B., Li X., Zuo A., Yang S., Ding S., Yang G. (2023). High-quality ultrathin Gd_2_O_2_S nanosheets with oxygen vacancy-decorated rGO for enhanced electromagnetic wave absorption. ACS Appl. Mater. Interfaces.

[110] Gahlawat R., Shukla R. (2025). Enhanced EMI shielding performance of CoFe_2−x_Dy_x_O_4_/polypyrrole nanocomposites in Ku-band. Surf. Interface.

[111] Mondal D., Bhattacharya D., Mondal T., Kundu M., Sarkar S., Mandal T.K., Paul B.K., Das S. (2023). Rare earth ion-infused α-MnO_2_ nano-rods for excellent EMI shielding efficiency: Experimental and theoretical insights. Sustain. Mater. Technol..

[112] Zboril R., Mashlan M., Petridis D. (2002). Iron(III) oxides from thermal processes synthesis, structural and magnetic properties, Mössbauer spectroscopy characterization, and applications. Chem. Mater..

[113] Raming T.P., Winnubst A.J.A., van Kats C.M., Philipse A.P. (2002). The synthesis and magnetic properties of nanosized hematite (α-Fe_2_O_3_) particles. J. Colloid Interface Sci..

[114] Morrish A.H., Freyhardt H.C. (1980). Morphology and Physical Properties of Gamma Iron Oxide. Growth and Properties.

[115] Uyama T., Mukai K., Yamada I. (2020). Facile and low-temperature synthesis of γ-Fe_2_O_3_ nanoparticles with thermally stable ferrimagnetism for use in magnetic recording tapes. ACS Appl. Nano Mater..

[116] Kelm K., Mader W. (2005). Synthesis and structural analysis of ϵ-Fe_2_O_3_. Z. Anorg. Allg. Chem..

[117] Azadmanjiri J., Hojati-Talemi P., Simon G.P., Suzuki K., Selomulya C. (2011). Synthesis and electromagnetic interference shielding properties of iron oxide/polypyrrole nanocomposites. Polym. Sci. Eng..

[118] Gupta A., Singh A.P., Varshney S., Agrawal N., Sambyal P., Pandey Y., Singh B.P., Singh V.N., Gupta B.K., Dhawan S.K. (2014). New insight into the shape-controlled synthesis and microwave shielding properties of iron oxide covered with reduced graphene oxide. RSC Adv..

[119] Dhawan S.K., Singh K., Bakhshi A.K., Ohlan A. (2009). Conducting polymer embedded with nanoferrite and titanium dioxide nanoparticles for microwave absorption. Synth. Met..

[120] Singh A.P., Mishra M., Sambyal P., Gupta B.K., Singh B.P., Chandra A., Dhawan S.K. (2014). Encapsulation of γ-Fe_2_O_3_ decorated reduced graphene oxide in polyaniline core–shell tubes as an exceptional tracker for electromagnetic environmental pollution. J. Mater. Chem. A.

[121] Rao B.V.B., Chengappa M., Kale S.N. (2017). Lightweight, flexible and thin Fe_3_O_4_-loaded, functionalized multi walled carbon nanotube buckypapers for enhanced X-band electromagnetic interference shielding. Mater. Res. Express.

[122] Liu Y., Song D., Wu C., Leng J. (2014). EMI shielding performance of nanocomposites with MWCNTs, nanosized Fe_3_O_4_ and Fe. Compos. B Eng..

[123] Li L.-Y., Li S.-L., Shao Y., Dou R., Yin B., Yang M.-B. (2018). PVDF/PS/HDPE/MWCNTs/Fe_3_O_4_ nanocomposites: Effective and lightweight electromagnetic interference shielding material through the synergetic effect of MWCNTs and Fe_3_O_4_ nanoparticles. Curr. Appl. Phys..

[124] Prasad J., Singh A.K., Shah J., Kotnala R.K., Singh K. (2018). Synthesis of MoS_2_-reduced graphene oxide/Fe_3_O_4_ nanocomposite for enhanced electromagnetic interference shielding effectiveness. Mater. Res. Express.

[125] Sambyal P., Dhawan S.K., Gairola P., Chauhan S.S., Gairola S.P. (2018). Synergistic effect of polypyrrole/BST/rGO/Fe_3_O_4_ composite for enhanced microwave absorption and EMI shielding in X-Band. Curr. Appl. Phys..

[126] Zhan Y., Wang J., Zhang K., Li Y., Meng Y., Yan N., Wei W., Peng F., Xia H. (2018). Fabrication of a flexible electromagnetic interference shielding Fe_3_O_4_@reduced graphene oxide/natural rubber composite with segregated network. J. Chem. Eng..

[127] Liu H., Liang C., Chen J., Huang Y., Cheng F., Wen F., Xu B., Wang B. (2019). Novel 3D network porous graphene nanoplatelets/Fe_3_O_4_/epoxy nanocomposites with enhanced electromagnetic interference shielding efficiency. Compos. Sci. Technol..

[128] Huangfu Y., Liang C., Han Y., Qiu H., Song P., Wang L., Kong J., Gu J. (2019). Fabrication and investigation on the Fe_3_O_4_/thermally annealed graphene aerogel/epoxy electromagnetic interference shielding nanocomposites. Compos. Sci. Technol..

[129] Liu Y., Lu M., Wu K., Yao S., Du X., Chen G., Zhang Q., Liang L., Lu M. (2019). Anisotropic thermal conductivity and electromagnetic interference shielding of epoxy nanocomposites based on magnetic driving reduced graphene oxide@Fe_3_O_4_. Compos. Sci. Technol..

[130] Liang C., Song P., Ma A., Shi X., Gu H., Wang L., Qiu H., Kong J., Gu J. (2019). Highly oriented three-dimensional structures of Fe_3_O_4_ decorated CNTs/reduced graphene oxide foam/epoxy nanocomposites against electromagnetic pollution. Compos. Sci. Technol..

[131] Yang J., Liao X., Li J., He G., Zhang Y., Tang W., Wang G., Li G. (2019). Light-weight and flexible silicone rubber/MWCNTs/Fe_3_O_4_ nanocomposite foams for efficient electromagnetic interference shielding and microwave absorption. Compos. Sci. Technol..

[132] Shu X., Fang B., Wu W., Song Y., Zhao Z. (2021). Acicular or octahedral Fe_3_O_4_/rice husk-based activated carbon composites through graphitization synthesis as superior electromagnetic wave absorbers. Compos.-A Appl. Sci..

[133] Shu X., Cheng J., Fang B., Wang J., Song Y., Lu W., Zhao Z. (2023). Morphology-dependent magnetic role of ZIFs in nitrogen-doped MXene as metallic conductor microwave absorber. J. Chem. Eng..

[134] Shu X., Yan S.C., Fang B., Song Y., Zhao Z. (2023). A 3D multifunctional nitrogen-doped RGO-based aerogel with silver nanowires assisted self-supporting networks for enhanced electromagnetic wave absorption. J. Chem. Eng..

